# Mild infection induced by low-dose LPS does not impair follicular development and is beneficial to pregnancy in mice

**DOI:** 10.3389/fvets.2022.1051433

**Published:** 2023-02-23

**Authors:** Yazhuo Du, Yutian Zeng, Shuo Li, Zhicheng Wang, Changqi Su, Shilin Zhang, Yan Ren, Tianzeng Song, Ming Zhang

**Affiliations:** ^1^College of Animal Science and Technology, Sichuan Agricultural University, Chengdu, China; ^2^Farm Animal Genetic Resources Exploration and Innovation Key Laboratory of Sichuan Province, Sichuan Agricultural University, Ya'an, China; ^3^Institute of Animal Science, Tibet Academy of Agricultural and Animal Husbandry Science, Lhasa, China

**Keywords:** lipopolysaccharide (LPS), follicle development, embryo implantation, pregnancy, mild infection

## Abstract

The reproductive tract is susceptible to infection by a variety of bacteria, which can impair ovarian and uterine function. However, there is little known about whether mild infection can harm follicle development and embryo implantation. Here our results showed that the immune response to a mild infection simulated by low-dose LPS induced inflammatory factor IL-1b expression and decreased MMP2 expression involved in embryo implantation. LPS treatment also inhibited the ovulation process and reduced litter weight. Despite the immune response and the disturbed ovulation induced by treatment with low-dose LPS, the overall result was beneficial to mouse pregnancy. This research provides the necessary foundation for exploring the effects of mild bacterial infection on ovarian and uterine function in mammals.

## 1. Introduction

The microbial community, metabolites and immune system coordinately regulate homeostasis and function in the reproductive tract (1). The reproductive tract of female animals is exposed to the external environment during the parturition period and is susceptible to bacteria. *Trueperella* spp., *Acinetobacter* spp., *Fusobacteria* spp., *Proteus* spp., *Prevotella* spp., and *Peptostreptococcus* spp., exist in the uterus and can cause metritis, endometritis and pyometra, which can inhibit embryo growth, and cause premature birth or miscarriage (2, 3).

Lipopolysaccharide (LPS), a component of Gram-negative bacterial cell walls, triggers an immune response by binding to the TLR4 receptor on the cell surface, activating myeloid differentiation factor (MyD88)-dependent and independent pathways, and inducing the secretion of inflammatory factors such as interleukin-1 beta (IL-1β), IL-10, and TNF-α (4). Hence, LPS can be used to simulate Gram-negative bacterial infection by inducing an appropriate immune response. Previous studies have confirmed that LPS could disturb follicular development (5), ovulation, embryonic development (6) and implantation, which can result in premature birth and miscarriage (7–9). However, although it is well known that inflammation triggered by LPS or bacterial infection contributes to reproductive disorders in mammals, we still know very little about the effect of the immune response on ovarian and uterine function, especially in mild infection. Mild infection refers to the condition in which the pathogen invading the body has weak virulence or the body possesses strong immunity (10, 11), so that an immune response is triggered, but the body shows no severe clinical symptoms (12). Therefore, in this study, we simulated mild infection with a low dose of LPS to determine its effects on follicular development, ovulation and uterine function.

## 2. Materials and methods

### 2.1. Animals

All experiments used 12-week-old ICR mice (Dashuo Co. Chengdu, China) that were housed in individual ventilated cages (IVC) under controlled 12-h light:12-h darkness) and temperature (18–24°C) conditions and provided with free access to water and food. The study and the animal treatment procedures were reviewed and approved by the Animal Ethical and Welfare Committee (AEWC) of Sichuan Agricultural University (No. DKYB20081003).

### 2.2. Experimental design

**Figure 1:**
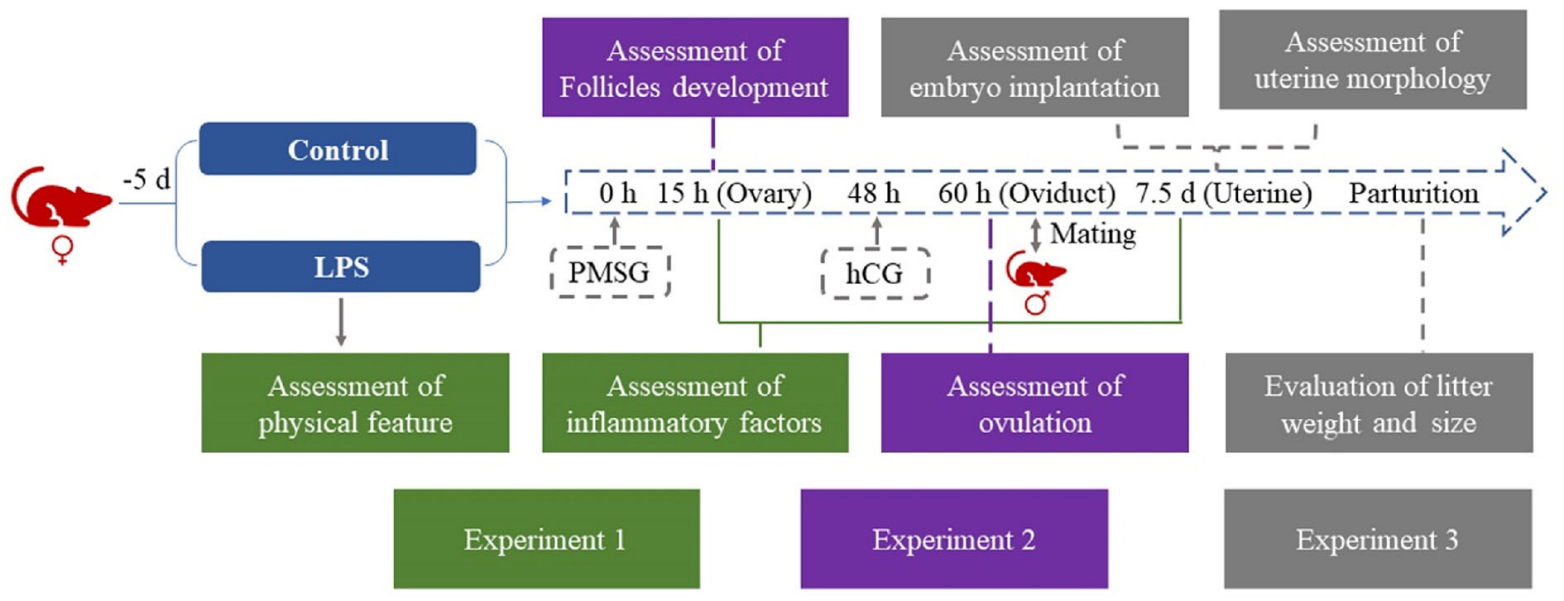
Flowchart of experimental design. LPS treatment was 50 μg/kg/d i.p. for 5 days in the LPS group.

Female ICR mice (110) were randomly divided into a control and an LPS group. Mice in the LPS group were injected with 50 μg/kg/d LPS i.p. for 5 days (8, 9), while control mice were injected with saline. The daily physical data on the animals during the experiment was recorded. Ovaries were collected after 15 h estrus synchronization for H&E staining and qRT-PCR. Cumulus-oocyte complexes (COCs) were collected from oviducts at 12 h after hCG treatment, and the number of MII oocytes was counted. In the next stage, female and male mice were caged together and successful mating was demonstrated by the presence of a vaginal plug on the following day. We counted the number of implantations and collected uterine horn samples during five days of gestation. Litter weight and size were recorded after parturition (Figure 1).

### 2.3. Food intake, weight, and rectal temperature measurement

For the mice injected with LPS or saline, the food consumption and body weight were recorded, along with the rectal temperature utilizing a soft tip electronic thermometer.

### 2.4. qRT-PCR

Total RNA was extracted from ovaries and uterine tissue using Trizol reagent (Invivogen, Shanghai, China) following the manufacturer's protocol (13). Relative mRNA expression levels (Table 1) were determined using the Takara PrimeScript^TM^ RT reagent kit with gDNA Eraser (Takara, Dalian, China), and the cDNAs were run on a Bio-Rad CFX-96 thermocycler (Bio-Rad, CA, USA). The primers used and the product size are shown in Table 1. The relative expression levels were calculated by the 2^−ΔΔCT^ method and the cytoskeletal protein, β- actin, was included as endogenous control to normalize the data.

**Table 1:** Primer information and product size for qRT-PCR.

**Gene symbol**	**Primer (5^′^-3^′^)**	**Product Size (nt)**	**Accession number**
IL-1β	F:TCATTGTGGCTGTGGAGAAGC	164	NM_008361.4
	R:AATGGGAACGTCACACACCAG		
BMP4	F:TCGTTACCTCAAGGGAGTGG	160	NM-007554.3
	R:GGCGACGGCAGTTCTTATTC		
GDF9	F:CAGTCCACCTGGAGGCCTTTA	125	NM_008110.2
	R:GAGCGGATGGCTTTCTGCCCT		
MMP2	F:CCAACTACGATGATGAC	233	NM_008610.3
	R:ACCAGTGTCAGTATCAG		
MMP9	F:GCCGACTTTTGTGGTCTTCC	80	NM_013599.5
	R:GGTACAAGTATGCCTCTGCCA		
TIMP2	F:CACGCTTAGCATCACCCAGA	77	NM_011594.3
	R:GACAGCGAGTGATCTTGCAC		
PRα	F:GCCTGGACAAAGAAGCACTG	114	NM_016783.4
	R:CGTGATGATACTTGAAAGTAGACTG		
β-ACTIN	F: CCACCATGTACCCAGGCATT	253	NM_007393.5
	R: AGGGTGTAAAACGCAGCTCA		

Tm: melting temperature was 60°C; IL-1β, interleukin-1 beta; BMP4, bone morphogenetic protein 4; GDF9, growth differentiation factor 9; MMP2, matrix metallopeptidase 2; MMP9, matrix metallopeptidase 9; TIMP2, tissue inhibitor of metalloproteinase 2; PRα, progesterone receptor α.

### 2.5. H&E staining

The ovary and uterine tissues were collected and fixed with 4% paraformaldehyde for 72 h at room temperature. After dehydration, the tissues were embedded, cut into 4 um-thick sections, and stained with hematoxylin and eosin (H&E). All images were obtained under an epifluorescence microscope (Olympus BX53, Tokyo, Japan). Pathological changes were observed and scored in ovarian and uterine tissues following published guidelines (14), antral follicles were counted and uterine physiological features were evaluated.

### 2.6. Statistical analysis

All data were reported as mean ± standard error. Statistical analysis was performed using SPSS (v.22.0, IBM, Chicago, IL, USA). Data of control and LPS groups were analyzed by Student's unpaired *t* test. The percentage of pregnant mice was compared between the two groups using the Fisher's exact test and *P* < 0.05 was considered statistically significant. Figures showing images for comparison were constructed with GraphPad Prism 8.0 software.

## 3. Results

### 3.1. Low-dose LPS induces mild infection and affects physical features and immune response in mice

**Figure 2:**
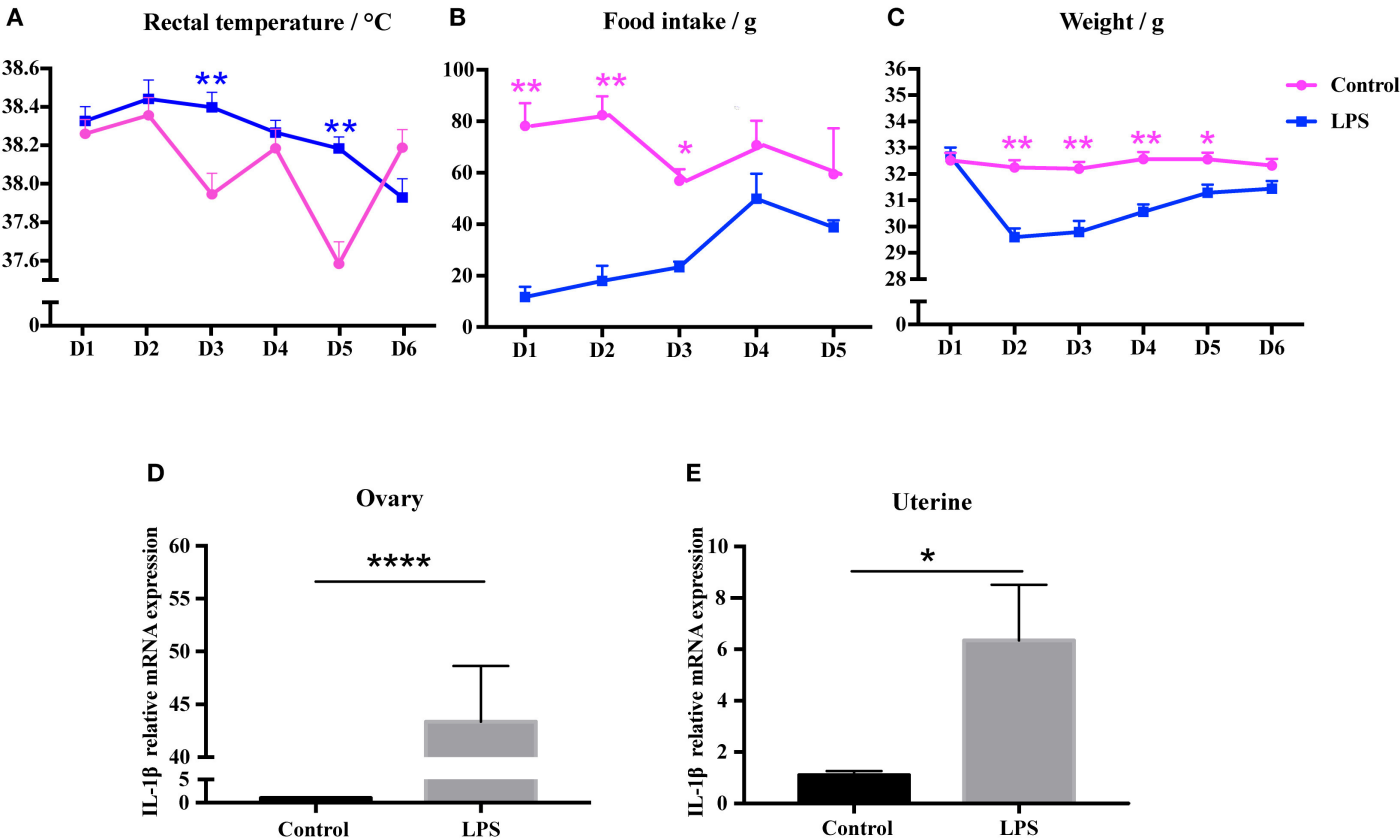
Low-dose LPS disturbs physiological features and induces an immune response in mice. **(A)** Rectal temperature during LPS injection (*n* = 24). **(B)** Food intake during LPS treatment (*n* = 24). **(C)** Weight during LPS injection (*n* = 24). **(D, E)** Relative IL-1β mRNA expression in ovary (*n* = 9) and uterus (*n* = 12).

We investigated whether repeated low-level injection of LPS affected the inflammatory response of mice, and found that mouse behavior and activity were normal, and no diarrhea was present. We measured food consumption, body weight, and rectal temperature. Unsurprisingly, the results showed that the average temperature was slightly above normal on day 3 and day 5 of LPS treatment (*P* < 0.01, Figure 2A), while food intake and body weight was significantly lower on days 1–3 compared to control mice (*P* < 0.05, Figures 2B, C). As an indicator of immune response we measured the relative expression level of IL-1β by qRT-PCR and showed that IL-1β mRNA level was significantly increased in ovary (*P* < 0.01, Figure 2D) and uterine tissues (*P* < 0.05, Figure 2E). These results suggest that low-dose LPS induced a mild infection, with some changes in physiological status but no severe symptoms.

### 3.2. Mild infection impairs the ovulation process, but does not affect follicular development

**Figure 3:**
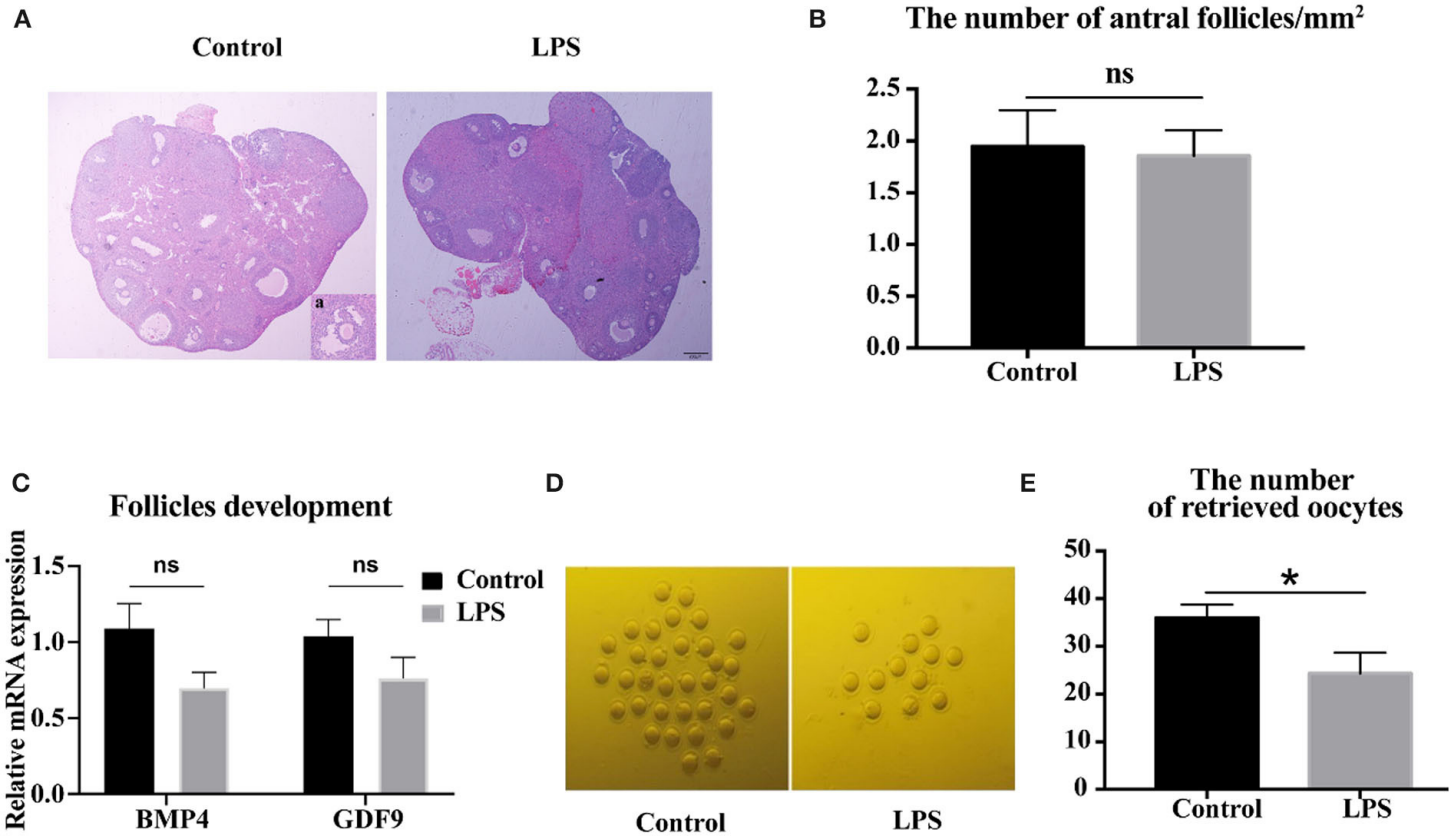
LPS impairs the ovulation processes, but does not affect follicular development. **(A)** H&E-stained ovarian tissue after 36 h synchronous estrus. Inset (a) shows representative image of antral follicle. Bar = 200 μm. **(B)** Number of antral follicles/mm^2^ after LPS treatment (*n* = 10). **(C)** Relative mRNA expression of BMP4 and GDF9 in ovaries (*n* = 9). **(D)** Representative images of MII oocytes in control and LPS group. **(E)** Number of oocytes retrieved from oviduct (control: LPS = 12: 10).

To determine whether mild infection affected development of antral follicles, ovarian structure was analyzed. The results showed no significant difference in the number of antral follicles between control and LPS groups (Figures 3A, B). The expression of growth differentiation factor 9 (GDF9) and bone morphogenetic protein 4 (BMP4), which play crucial roles in primary follicular development, were not altered (*P* > 0.05, Figure 3C) after LPS treatment. However, LPS did significantly decrease the ovulation rate (*P* < 0.05, Figures 3D, E). Overall, the mild infection had a detrimental influence on ovulation stage but not follicle development.

### 3.3. Effect of mild infection on embryo implantation and reproductive rate

**Figure 4:**
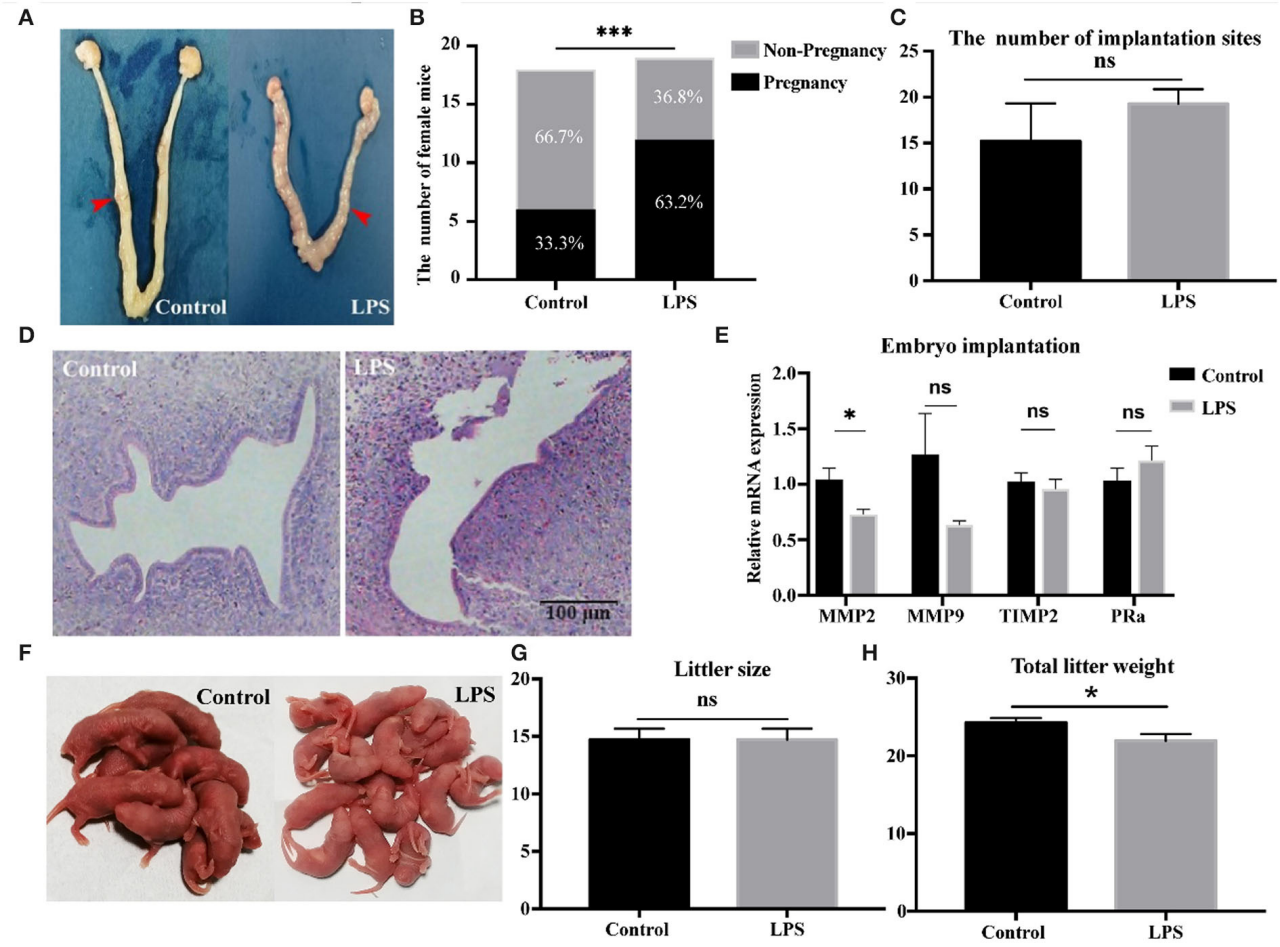
Mild infection increased the pregnancy rate in mice. **(A)** Representative images of the uterus at 5 days gestation. **(B)** Number of pregnant mice at 5 days gestation (control : LPS = 18: 19). **(C)** Number of implantation sites (control: LPS = 6: 12). Average no. of implantation sites = $\frac{number\;of\;implantation\;sites\;in\;bilateral\;uherine\;horn}{number\;of\;pregnant\;mice}$. **(D)** Representative H&E images of uterus at 5 days gestation. **(E)** Relative mRNA expression of MMP2, MMP9, TIMP2, PRα in uterine tissues (*n* = 12). **(F)** Representative images of pups delivered by saline-treated controls and LPS-treated animals. **(G, H)** showed the average number of pups and litter weight delivered by control and LPS group (control: LPS = 12: 10). * *p* < 0.05; ***p < 0.001.

To determine if mild infection inhibited embryo implantation, we counted the implantation sites 5 days after observing vaginal plugs (Figure 4A). Results showed a significant difference in pregnancy rate between control and LPS groups (*P* < 0.001, Figure 4B), but no difference in the average number of embryo implantation sites (*P* > 0.05, Figure 4C) and tissue structure of endometrium (Figure 4D). We measured relative mRNA expression of matrix metallopeptidase 2 (MMP2), matrix metallopeptidase 9 (MMP9) and tissue inhibitors of metalloproteinase 2 (TIMP2), an inhibitor of MMP2, and found that MMP2 expression was significantly decreased (*P* < 0.05, Figure 4E) in the LPS group, but MMP9 and TIMP2 expression showed no difference (Figure 4E). We also measured the mRNA expression of PRα, and found no difference between the groups (Figure 4E). To determine whether mild infection disturbed the final reproductive performance, we recorded the litter size and weight. Mild infection had no influence on litter size (*P* > 0.05, Figure 4G), but litter weight was significantly lower (*P* < 0.05, Figures 4F, H). Thus, mild infection had a positive effect on the pregnancy rate, although relative gene expression, and litter weight were significantly decreased.

## 4. Discussion

The reproductive tract is susceptible to infection by Gram-negative bacteria, which can seriously damage mammalian reproduction. Mild infection refers to the condition where an infection with a pathogenic microorganism induces an immune response, but produces no severe clinical symptoms. In the present study, the novel result suggested the mild infection or low-level immune response is beneficial to pregnancy.

In mice, we determined the dosage regimen through relevant references and preliminary experiments, we wanted to establish a low-level infection that lasted a few days, so finally we decided to use 50 μg/kg/d LPS to induce mild infection. At first, our results were similar to those in Arsenault's study, which showed that food intake and body weight of female mice decreased after LPS injection (15), and meanwhile, mice did not show diarrhea, depression and other abnormal behaviors. In addition, IL-1β, an important indicator of inflammation, was also significantly increased in ovaries and uterus after LPS injection. Taken together, these results indicated that mild infection was established through repeated low-level injection of LPS.

Studies have shown that BMP4 and GDF9 play a crucial role in the initiation of primordial follicular development and the growth of primary follicles, respectively (16, 17). Previous research has shown that LPS can disturb the primordial follicle pool, promote primordial follicle development, and accelerate follicular atresia (5). Some studies showed that LPS activated the NF-κB signal pathway, then inhibited the secretion of estrogen in mouse granulosa cells, and further decreased antral follicles and ovulation (18). LPS at high levels in the follicular fluid inhibited steroid production, further affecting dominant follicle development (19, 20). However, our results showed that the number of antral follicles did not change after low-level treatment with LPS, and differences in expression levels of BMP4 and GDF9 mRNA were not significant. That effect may be due to insufficient stimulation by low levels of LPS or the development of tolerance to LPS in mice. Similarly, chronic inflammation induced by LPS had no detrimental effect on follicle development, or the size of dominant follicles and estrogen and progesterone levels in dairy cows (21). Our experiment and the chronic inflammation studies imply that low-dose LPS doesn't impair on follicle development and the genes related to follicle growth. However, LPS disturbed GnRH and LH secretion by the hypothalamic-pituitary-ovarian axis, and the endocrine disorders could further impair ovulation (22). Our results also showed a significant decrease in ovulation rate. Therefore, the mild infection or the chronic inflammation might only impaired ovulation, but did not affect antral follicular development.

Chronic endometritis triggering by Gram-negative bacteria is often seen as the reason for implantation failure and early embryo loss. LPS has a detrimental effect on embryo implantation (7, 23), but some studies pointed out that a low level of LPS was beneficial for implantation because the process is similar to an immune response (24). Interleukins regulate the proliferation and maturation of natural killer cells, and T and B cell function, inhibit the secretion of immune tolerance-related antibodies to support the trophoblast invasion, decidua formation, embryo attachment to the endometrium, spiral arterial remodeling and placenta formation during embryo implantation (25). Our results showed a significant increase in IL-1β expression levels after LPS-treatment, and the percentage of pregnant mice also increased. At the same time, we observed that there was no significant loss of implantation sites after LPS-treatment, which supports the hypothesis that mild infection benefits embryo implantation and improves pregnancy rate. However, high IL levels are likely to cause cytotoxicity, damage the immune microenvironment in the endometrium, trigger immune rejection, and ultimately cause pregnancy failure and miscarriage (26–28).

MMPs and TIMPs are critical for embryo invasion during the window of implantation (29). Abnormal expression of MMP2 and MMP9 results in vasodilation, and placental and uterine dilation dysfunction (30). Decreasing MMP2 and MMP9 impairs spiral artery remodeling and causes initial pathological symptom of preeclampsia during early gestation. Vasoactive factors are released by increasing MMP2 and MMP9, which further increases the risk of hypertension during late gestation (31). Previous research demonstrated that IL-1β activated MMP9 expression and further regulated embryo invasion (32). MMP9 downregulated IL-1b expression (33). Overall, the expression levels of MMP2 and MMP9 can reflect embryo implantation to a certain extent. Our experiments showed that LPS decreased the MMP2 expression level. Other studies showed that increasing MMP9 and the MMP2/TIMP 2 ratio increased the risk of miscarriage (34). In addition, the progesterone receptor (PR) plays an important role in the establishment of endometrial receptivity during the implantation window (35), implantation, decidualization, and glandular development via paracrine secretion (36). LPS can significantly reduce the expression of PR during the ‘window' period (37). Aisemberg's experiments indicated that LPS decreased PR protein level (38), however, our results showed that low levels of LPS did not affect PRα expression, and the reason may be that low levels of LPS were not sufficient to impair embryo implantation, or because the uterus became tolerant to multiple treatments with low dose LPS during implantation. Taken together, although LPS did induce an immune response, mild infection had no apparent negative effect on embryo implantation and appeared to be beneficial for pregnancy. The effect and specific mechanism of different degrees of inflammatory infection on embryo implantation need to be further studied.

Previous studies showed that LPS resulted in premature birth and decreased the litter size (12). However, our results showed mild infection with LPS did not cause miscarriage or decrease the litter size. While litter weight was significantly decreased, this may be because the intrauterine growth of embryos was hindered in late gestation. Therefore, mild infection slightly disturbed the intrauterine growth of fetal in the process of pregnancy but not the pregnancy rate.

Based on these previous and our studies imply different levels of immune response induced by different degrees of infection or inflammation have different effects on the reproductive function of female animals at different stages. Therefore, it is necessary to further study the effects of different degrees of inflammation on female genital tract at different reproductive stages.

## 4. Conclusions

Mild infection established by low levels of LPS disturbed certain physiological aspects and induced an immune response without severe clinical symptoms in mice. However, mild infection affected ovulation and the litter size, but did not impair the follicle development and implantation. In future, more research is essential to further clarify the mechanism of mild infection on the ovary and uterine function.

## Data availability statement

The original contributions presented in the study are included in the article/supplementary material, further inquiries can be directed to the corresponding author.

## Ethics statement

The animal study was reviewed and approved by the Animal Ethical and Welfare Committee (AEWC) of Sichuan Agricultural University.

## Author contributions

MZ conceived and designed the experiments. YD, SL, and ZW performed the experiments and manuscript drafting. YZ participated in manuscript editing. CS, SZ, YR, and TS take part in important discussion. All authors have read and agreed to the current version of the manuscript.

## References

[1] Li H Zang Y Wang C Li H Fan A Han C. The interaction between microorganisms, metabolites, and immune system in the female genital tract microenvironment. Front Cell Infect Microbiol. (2020) 10:609488. 10.3389/fcimb.2020.609488 33425785 PMC7785791

[2] Turner ML Healey GD Sheldon IM. Immunity inflammation in the uterus. Reprod Domest Anim. (2012) 47:402–9. 10.1111/j.1439-0531.2012.02104.x 22827398

[3] Rosales E Ametaj B. Reproductive tract infections in dairy cows: can probiotics curb down the incidence rate? Dairy. (2021) 2:40–64. 10.3390/dairy2010004

[4] Khan KN Kitajima M Hiraki K Fujishita A Sekine I Tadayuki T. Toll-like receptors in innate immunity: role of bacterial endotoxin and toll-like receptor 4 in endometrium and endometriosis. Gynecol Obstet Invest. (2009) 68:40–52. 10.1159/000212061 19365133

[5] Bromfield Sheldon IM. Lipopolysaccharide reduces the primordial follicle pool in the bovine ovarian cortex ex vivo and in the murine ovary *in vivo*. Biol Reprod. (2013) 88:98. 10.1095/biolreprod.112.106914 23515670

[6] Heidari M Kafi M Mirzaei A Asaadi A Mokhtari A. Effects of follicular fluid of preovulatory follicles of repeat breeder dairy cows with subclinical endometritis on oocyte developmental competence. Anim Reprod Sci. (2019) 205:62–9. 10.1016/j.anireprosci.2019.04.004 31005360

[7] Deb K Chaturvedi MM Jaiswal YK. Gram-negative bacterial LPS induced poor uterine receptivity and implantation failure in mouse: alterations in IL-1beta expression in the preimplantation embryo and uterine horns. Infect Dis Obstet Gynecol. (2005) 13:125–33. 10.1080/10647440500147885 16126496 PMC1784569

[8] Toyama RP Xikota C Schwarzbold ML Frode T Buss Z Nunes J. Dose-dependent sickness behavior, abortion and inflammation induced by systemic LPS injection in pregnant mice. Matern Fetal Neonatal Med. (2015) 28:426–30. 10.3109/14767058.2014.918600 24824102

[9] Lee A Kandiah N Karimi K Clark D Ashkar A. Interleukin-15 is required for maximal lipopolysaccharide-induced abortion. Leukoc Biol. (2013) 93:905–12. 10.1189/jlb.0912442 23505315

[10] Chiara P Lyndal H Sara P Rebecca L Larkin R Higgins DP. Chlamydia pecorum Infection in the male reproductive system of koalas (*Phascolarctos cinereus*). Vet Pathol. (2019) 56:300–6. 10.1177/0300985818806963 30381016

[11] Zhou Y Niu XD Ding YD Qian Z Zhao B. Prevalence of recessive infection of pathogens of hand, foot, and mouth disease in healthy people in China. Medicine. (2021). 100:e24855. 10.1097/MD.0000000000024855 33607859 PMC7899851

[12] Liu S Shi Y Liu C Zhang M Zuo ZC Zeng CJ. The upregulation of pro-inflammatory cytokines in the rabbit uterus under the lipopolysaccaride-induced reversible immunoresponse state. Animal Reproduct Sci. (2017) 176:70–77 10.1016/j.anireprosci.2016.11.012 27916460

[13] Zeng YT Wang C Zhang Y Xu L Zhou GB Zeng CJ. Improvac immunocastration affects the development of thigh muscles but not pectoral muscles in male chickens. Poult Sci. (2020) 99:5149–57. 10.1016/j.psj.2020.06.040 32988554 PMC7598331

[14] Chen L Lei L Chang X Li Z Lu C Zhang X. Mice deficient in MyD88 Develop a Th2-dominant response and severe pathology in the upper genital tract following Chlamydia muridarum infection. Immunol. (2010) 184:2602–10. 10.4049/jimmunol.0901593 20124098

[15] Arsenault D St-Amour I Cisbani G Rousseau LS Cicchetti F. The different effects of LPS and poly I:C prenatal immune challenges on the behavior, development and inflammatory responses in pregnant mice and their offspring. Brain Behav Immun. (2014) 38:77–90. 10.1016/j.bbi.2013.12.016 24384468

[16] Nilsson EE Skinner MK. Bone morphogenetic protein-4 acts as an ovarian follicle survival factor and promotes primordial follicle development. Biol Reprod. (2003) 69:1265–72. 10.1095/biolreprod.103.018671 12801979

[17] Nilsson EE Skinner MK. Growth differentiation factor-9 stimulates progression of early primary but not primordial rat ovarian follicle development. Biol Reprod. (2002) 67:1018–24. 10.1095/biolreprod.101.002527 12193416

[18] Guan HY Xia HX Chen XY Wang L Tang Z Zhang W. Toll-like receptor 4 inhibits estradiol secretion *via* NF-κB signaling in human granulosa cells. Front Endocrinol (Lausanne). (2021) 12:629554. 10.3389/fendo.2021.629554 33776924 PMC7995891

[19] Magata F Horiuchi M Echizenya R Miura R Chiba S Matsui M. Lipopolysaccharide in ovarian follicular fluid influences the steroid production in large follicles of dairy cows. Anim Reprod Sci. (2014) 144:6–13. 10.1016/j.anireprosci.2013.11.005 24321186

[20] Forrest K K Flores V V Gurule S C Navarro S Shuster CB Gifford C. Effects of lipopolysaccharide on follicular estrogen production and developmental competence in bovine oocytes. Anim Reprod Sci. (2022) 237:106927. 10.1016/j.anireprosci.2022.106927 35074697 PMC8928215

[21] Dickson M Kvidera SK Horst EA Wiley CE Mayoga EJ Ydstie J. Impacts of chronic and increasing lipopolysaccharide exposure on production and reproductive parameters in lactating Holstein dairy cows. Dairy Sci. (2019) 102:3569–83. 10.3168/jds.2018-15631 30738665

[22] Izvolskaia MS Tillet Y Sharova VS Voronva S Zakharova L. Disruptions in the hypothalamic-pituitary-gonadal axis in rat offspring following prenatal maternal exposure to lipopolysaccharide. Stress. (2016) 19:198–205. 10.3109/10253890.2016.1149695 26941006

[23] Moustafa S Joseph DN Taylor RN Whirledge S. New models of lipopolysaccharide-induced implantation loss reveal insights into the inflammatory response. Am Reprod Immunol. (2019) 81:e13082. 10.1111/aji.13082 30604526 PMC6433508

[24] Dekel N Gnainsky Y Granot I Taylor R Whirledge S. Inflammation and implantation. Am Reprod Immunol. (2010) 63:17–21. 10.1111/j.1600-0897.2009.00792.x 20059465 PMC3025807

[25] Pantos K Grigoriadis S Maziotis E Pistola K Xystra P Pantou S. The role of interleukins in recurrent implantation failure: a comprehensive review of the literature. Int Mol Sci. (2022). 23:4. 10.3390/ijms23042198 35216313 PMC8875813

[26] Chen X Mariee N Iang L Liu Y Wang CC Liu T. Measurement of uterine natural killer cell percentage in the periimplantation endometrium from fertile women and women with recurrent reproductive failure: establishment of a reference range. Am Obstet Gynecol. (2017) 217:680.e1–e6. 10.1016/j.ajog.2017.09.010 28935491

[27] Kolanska K Bendifallah S Cohen Placais L Sellert L Johanet C. Unexplained recurrent implantation failures: Predictive factors of pregnancy and therapeutic management from a French multicentre study. Reprod Immunol. (2021) 145:103313. 10.1016/j.jri.2021.103313 33774529

[28] Lédée N Petitbarat M Chevrier L Vitoux D Vezmar K Rahmati M. The uterine immune profile may help women with repeated unexplained embryo implantation failure after *in vitro* fertilization. Am Reprod Immunol. (2016) 75:388–401. 10.1111/aji.12483 26777262 PMC4849202

[29] Chen R Khalil A. Matrix metalloproteinases in normal pregnancy and preeclampsia. Prog Mol Biol Transl Sci. (2017) 148:87–165. 10.1016/bs.pmbts.2017.04.001 28662830 PMC5548443

[30] Nikolov A Popovski N. Role of gelatinases MMP-2 and MMP-9 in healthy and complicated pregnancy and their future potential as preeclampsia biomarkers []. Diagnostics (Basel). (2021) 11:3. 10.3390/diagnostics11030480 33803206 PMC8001076

[31] Espino YSS Flores-Pliego A Espejel-Nuñez A Bastidas D Ortego F Clavellna V. New insights into the role of matrix metalloproteinases in preeclampsia. Int Mol Sci, (2017) 18:7. 10.3390/ijms18071448 28726716 PMC5535939

[32] Librach CL Feigenbaum SL Bass KE Verastas N Sadovsky Y. Interleukin-1 beta regulates human cytotrophoblast metalloproteinase activity and invasion *in vitro*. Biol Chem. (1994) 269:17125–31. 10.1016/S0021-9258(17)32529-2 8006017

[33] Ito A Mukaiyama A Itoh Y Nagase H Thogersen IB Enghild JJ. Degradation of interleukin 1beta by matrix metalloproteinases. Biol Chem. (1996) 271:14657–60. 10.1074/jbc.271.25.14657 8663297

[34] Nissi R Talvensaari-Mattila A Kotila V Niniimaki M Jarvela I Hujanen T. Circulating matrix metalloproteinase MMP-9 and MMP-2/TIMP-2 complex are associated with spontaneous early pregnancy failure. Reprod Biol Endocrinol. (2013) 11:2. 10.1186/1477-7827-11-2 23320481 PMC3566964

[35] Mulac-ericevic B Mullinax RA Demayo F Lydon JP Conneely OM. Subgroup of reproductive functions of progesterone mediated by progesterone receptor-B isoform. Science (New York, NY). (2000) 289:1751–4. 10.1126/science.289.5485.1751 10976068

[36] Wetendorf M Demayo F. The progesterone receptor regulates implantation, decidualization, and glandular development *via* a complex paracrine signaling network. Mol Cell Endocrinol. (2012) 357:108–18. 10.1016/j.mce.2011.10.028 22115959 PMC3443857

[37] Agrawal V Jaiswal MK Jaiswal YK. Lipopolysaccharide-induced modulation in the expression of progesterone receptor and estradiol receptor leads to early pregnancy loss in mouse. Zygote (Cambridge, England). (2013) 21:337–44. 10.1017/S0967199412000330 22809764

[38] Aisemberg Vercelli CA Bariani MV Billi SC Wolfson M Franchi AM. Progesterone is essential for protecting against LPS-induced pregnancy loss. LIF as a potential mediator of the anti-inflammatory effect of progesterone. PLoS ONE. (2013) 8:e56161. 10.1371/journal.pone.0056161 23409146 PMC3567061

